# Associated factors of doctor visits made by urban-dwelling older adults in Sri Lanka: an application of Anderson’s model of health service utilization

**DOI:** 10.1186/s12877-022-03249-3

**Published:** 2022-07-11

**Authors:** Bimba I. Wickramarachchi, Sidiah J. Siop, Bilesha Perera

**Affiliations:** 1 Department of Nursing, Faculty of Medicine and Heath Sciences, Universiti Malaysia Sarawak, Kota Samarahan, Sarawak Malaysia; 2 Department of Nursing, Faculty of Allied Health Sciences, University of Ruhuna, Galle, Sri Lanka; 3 Department of Community Medicine, Faculty of Medicine, University of Ruhuna, Galle, 80000 Sri Lanka

**Keywords:** Doctor visits, Older adults, Self-rated health, Physical activity, Sri Lanka

## Abstract

**Background:**

Although universal free healthcare is available for all Sri Lankan citizens, older adults face somewhat unique obstacles when utilizing available healthcare services. The aim of this study was to examine some vital predisposing, enabling, and need factors associated with doctor visits made by urban-dwelling older adults in Sri Lanka.

**Methods:**

A representative sample of 880 urban-dwelling older adults (aged 60 years and above) was surveyed using an interviewer-administered questionnaire. Number of doctor visits, self-rated health, physical activity, and socio-demographic and self-report health conditions were collected. The data were analyzed using chi-squared tests and multinomial logistic regression.

**Results:**

Participants’ mean age was 70.01 (± 6.02) years. The majority was women (75.0%). The mean number of doctor visits was 6.77 (± 5.92) per year. Nearly half of the participants (47.0%) had made, on average, at least one doctor visit per month. Older men and those of aged 80 years and above were the least likely to make frequent doctor visits. Participants who were physically active and who rated their health as poor were more likely to make frequent doctor visits after adjustment for age, gender, and educational level.

**Conclusions:**

Doctor visits made by Sri Lankan older adults are satisfactory. The factors that best explain high frequency of doctor visits by older adults are female gender, younger age, higher physical activity and poor self-rated health. Attention should be paid to examine possible accessible and affordable issues related to doctor visits by bedridden or physically dependent older adults in advanced age categories.

**Supplementary Information:**

The online version contains supplementary material available at 10.1186/s12877-022-03249-3.

## Background

Sri Lanka, a middle-income country in South Asia, has one of the fastest aging populations in the region [1]. Population aging in Sri Lanka is not parallel to its economic growth and the majority of older adults in Sri Lanka do not have any social security benefits. The situation would create devastating health and economic consequences in the near future if necessary policy and corrective actions are not taken in time [1, 2]. Geriatric healthcare in the country is still in its infancy and the rates of healthcare utilization by older adults found to be low [3–5].

Individual decision-making regarding medical consultations or treatment is a complex process [6–8]. Gender, age, non-proximity of services, self-perception about health status, personal attributes, decision-making power, knowledge of health information and physical dependence have been suggested as vital factors associated with the use of healthcare facilities by older adults [5–8]. Although economic status is a strong factor associated with the use of healthcare, universal free health care available in Sri Lanka may have influenced this association significantly in the Sri Lankan context. It should be noted, however, that out-of-pocket expenses for medical treatments, particularly for medicines and other pharmaceutical items, are considerable [3], and access to care in the public health sector in the country is pro-poor [3].

Self-rated health (SRH) reflects an individual’s evaluation of their own health status based on their attitude and beliefs regarding the biological, psychological, and social dimensions of health [8, 9]. Barsky et al. [10] listed patients’ medical morbidity, psychiatric comorbidity, and functional disability as factors that may influence SRH. Self-rated health, has been applied in many studies to investigate healthcare seeking status and healthcare utilization [11].

Physical activity participation has been found to be a mobilizer of healthcare utilization among older adults [12–14]. Further, studies have demonstrated a positive association between physical activity and SRH. In Sri Lanka, owing to economic and ecological pressures, urban life involves multifaceted barriers to physical activity, such as congested and noisy environments, lack of facilities for leisure activities, and poor social interactions [15–17]. Such barriers subsequently would affect health of the urban population negatively forcing them to seek medical treatments.

Anderson’s well-known conceptual framework for healthcare utilization is composed of predisposing, enabling, and need factors that influence healthcare seeking behavior of people [8]. Accordingly, predisposing factors such as age, gender, and education; enabling factors such as income, health insurance, and physical fitness; and need factors such as illness severity and SRH together help determine healthcare utilization behavior more comprehensively in older populations. Healthcare utilization includes doctor visits, use of mobile health services, use of counseling services, use of paramedical services and many more. Healthcare utilization behavior, specifically on chronic diseases, of older adults is a health priority given that Sri Lankan population is rapidly aging. Personal, socio-cultural and system barriers of healthcare utilization in primary healthcare section in older adults in the country is simply not researched. Such information is essential on achieving holistic, team based care to coordinate limited resources available for geriatric healthcare in the country. This study sought to identify the pattern of doctor visits, a vital component in healthcare utilization made by older urban-dwelling Sri Lankans and to investigate several predisposing, enabling, and need factors that determine such utilization patterns.

## Methods

### Study design, setting, and participants

A cross-sectional survey was conducted in a sample of 880 urban-dwelling older adults (aged 60 years and above) from two Divisional Secretariat (DS) areas in Colombo district through multi-stage cluster sampling. Each of these DS divisions is further divided into several Grama Niladari (GN) divisions and each GN division has further been divided into population clusters. There are approximately 25 families live in each cluster. Five GN divisions from each DS areas selected were randomly selected and a total of 44 clusters were selected randomly from those 10 GN divisions. Data were not collected from 2 clusters out of 44 due to time and cost limitations. Although, originally we were planned to recruit 1100 older adults, we were able to recruit only 880 due to this. Response rate was 87%. However, the sample that we collected fully represents urban-dwelling older adults in Sri Lanka.

The inclusion criteria for participants were 1) aged 60 years and above; 2) able to communicate in Sinhala; and 3) leading a normal daily life. Hospitalized older adults were excluded. Older adults who were severely sick or had a poor cognitive state (Mini-Mental State Examination score < 17) with communication difficulties were also excluded. Older adults who migrated to other districts during the data collection period, as well as those who were homeless, were excluded. Data analysis was done using 880 participants.

### Measurements

#### Outcome variable

##### Frequency of doctor visits

Frequency of doctor visits for a medical reason in the previous 12 months was determined using self-reported data and medical records, and grouped into four categories: 0 visits, 1–5 visits, 6–11 visits, and 12 or more visits.

#### Independent variables

##### Self-rated health

Self-rated health was assessed using a single item: “How do you rate your health status?” The response options ranged from “very good” to “very poor.” In this study, responses were categorized into three categories as poor/very poor, fair, and very good/good by combining responses.

##### Physical activity

Physical activity was assessed using the Global Physical Activity Questionnaire version 2, which has been validated for use in older adults in Sri Lanka [18, 19]. This 16-item questionnaire assesses the levels (inactive or sufficiently active), domains (activity at work, active transport, and leisure time), and intensity of physical activity participation. Participants with more than 1500 MET min/week of physical activity were considered sufficiently active and those with less than 1500 MET min/week were considered inactive.

##### Socio-demographic variables

Data on age, gender, marital status, family income, and educational level were collected. Age was categorized as “young-old” (60–69 years), “middle-old” (70–79 years), and “oldest-old” (80 years and above). Educational level was categorized into “below primary education,” “secondary education,” and “tertiary education.” Family income (per month), in Sri Lankan Rupees (SLR), was categorized into “low-income” (less than 10,000 SLR), “middle-income” (10000–100,000 SLR), and “high-income” (more than 100,000 SLR). Marital status data were dichotomized as “married and living with spouse” and “other.”

##### Number of chronic diseases

Number of chronic diseases was determined by participants’ self-reports and verified through medical records and the subsequent interview. The presence of 18 most prevalent chronic conditions found in this target population was determined through a dichotomous question (yes or no). Based on their total number of chronic conditions, participants were further categorized into one of three groups: “no chronic diseases,” “one to two chronic diseases,” and “more than two chronic diseases.”

### Data collection procedure

A researcher-administered questionnaire was used for data collection. Eligible older adults were invited to participate in the study through a telephone call or home visit. Those who were willing to participate were screened for cognitive impairment using the Mini-Mental State Examination. Eligible participants were asked to be available for the study in the home setting at a convenient time. They were also asked to provide their medical records during the face-to-face interview, conducted in the local language, Sinhala. Data collection was conducted by the principal investigator, a trained nurse, with support from trained research assistants. The principal investigator double checked all the records and clarifications were sought from the participants to ensure reliability and validity of the data. The survey was conducted from August to December 2018.

### Ethical considerations

Participation was voluntary and subsequent to the provision of written informed consent. The data were anonymized and participants were assured of confidentiality. Ethical approval was obtained from the Ethics Review Committees of the Faculty of Medicine and Health Sciences, Universiti Malaysia Sarawak, Sarawak, Malaysia [(UNIMAS/NC-21.02/03–02 Jld.2 (94)] and the Faculty of Allied Health Sciences, University of Ruhuna, Galle, Sri Lanka (12. 07. 2018:3.1). Data collection tool was used with permission from the relevant copyright authors. All methods were carried out in accordance with relevant guidelines and regulations under ethics approval and consent to participate.

### Statistical analysis

The data were analyzed using SPSS Statistics, version 25.0 (IBM Corp., Armonk, NY, USA). Descriptive data were presented as numbers and percentages. Associations of observed factors with the frequency of doctor visits were determined by chi-square tests. Bivarible multinomial logistic regression was used to identify variables that were significantly associated with the frequency of doctor visits. This study used multinomial logistic regression instead of ordinal logistic regression as the data did not meet the parallel lines assumption. The data set met the requirements for multinomial logistic regression.

All significant factors in the bivariate model were separately loaded to three different multivariable multinomial logistic models to determine the associations of need, enabling, and predisposing factors with healthcare utilization (number of doctor visits in the past 12 months). Self-rated health (need factor) was loaded to the first model, followed by physical activity (enabling factor) to the second model, and the predisposing factors (age, gender, and educational level) to the third model. Outcomes were presented for each of the three categories: “1–5 doctor visits in the last 12 months,” “6–11 doctor visits in the last 12 months,” and “12 or more doctor visits in the last 12 months” compared to no doctor visits. The results were presented as odds ratios (ORs) with 95% confidence intervals (CIs). The significance level was set at *p* < 0.05.

## Results

### Description of study population and independent variables

The mean age of the participants was 70.20 years (*SD* = 6.02), 75.0% were women and 61.8% were living with their spouse. Although the proportion of older men in the sample was low, data was collected from 220 older men, which is quite adequate to make valid conclusions. About 86.8% of the respondents had completed at least secondary level education. About two-third of the participants were in poor income category (monthly income less 10,000 SLR (USD 50)). About 60% of the participants reported having three or more chronic health conditions. Women had a slightly higher mean body mass index (BMI) value than men (26.51 vs 24.03, *p* < 0.05). Twenty percent of the participants had an inactive life style and 32.5% reported that their SRH was either very good or good.

### Frequency of doctor visits

About 80.0% of the participants had made at least one doctor visit in the last year, and nearly half (47.0%), on average, made at least one doctor visit per month. The mean number of doctor visits was 6.77 per year (*SD* ± 5.92).

### Correlates of healthcare utilization

#### Predisposing factors

Age, gender, and educational level were significantly associated with the frequency of doctor visits. The oldest-old were the most likely to make the fewest doctor visits. About 43.0% of those aged 80 years and above had not made a doctor visit in the last year compared to 22.7% in the age group of 70–79 years and 31.0% in the age group of 60–69 years (Table 1).

**Table 1:** Distribution of socio-demographic characteristics and frequency of doctor’s visits of the participants (*n* = 880)

Covariates	n %	Frequency of doctor visits /year				***P***
		None n %	1 to 5 times n %	6 to 11 times n %	12 or more n %	
**Total**	880	247 (28.1)	153 (17.4)	66 (7.5)	414 (47.0)	
**Age (years)**						
60–69	437 (49.7)	135 (30.9)	84 (19.2)	24 (5.5)	194 (44.4)	.012
70–79	387 (44.0)	88 (22.7)	62 (16.0)	40 (10.4)	197 (50.9)	
80 and over	56 (6.3)	24 (42.9)	7 (12.5)	2 (3.5)	23 (41.1)	
**Gender**						
Male	220 (25.0)	65 (29.5)	55 (25.0)	26 (11.8)	74 (33.6)	.000
Female	660 (75.0)	182 (27.6)	98 (14.8)	40 (6.2)	340 (51.5)	
**Marital status**						
Married	544 (61.8)	156 (28.7)	93 (17.1)	38 (7.0)	257 (47.2)	.852
Unmarried/widow/Separated	336 (38.2)	91(27.1)	60 (17.9)	28 (8.3)	157 (46.7)	
**Education**						
Primary education	116 (13.2)	40 (34.5)	14 (12.1)	2 (1.7)	60 (51.7)	.000
Secondary education	675 (76.7)	189 (28.0)	112 (16.6)	53 (7.9)	321 (47.6)	
Tertiary education	89 (10.1)	18 (20.2)	27 (30.3)	11 (12.4)	33 (37.1)	
**Family income**						
Low income	556 (63.2)	162 (29.1)	96 (17.3)	37 (6.7)	261 (46.9)	.211
Middle income	196 (22.3)	45 (23.0)	41 (20.9)	20 (10.2)	90 (45.9)	
High income	128 (14.5)	40 (31.3)	16 (12.5)	9 (7.0)	63 (49.2)	
**SRH**						
Very good/good	286 (32.5)	114 (39.9)	38 (13.3)	16 (5.6)	118 (41.2)	.001
Fair	394 (44.8)	111 (28.2)	91 (23.1)	38 (9.6)	154 (39.1)	
Poor	200 (22.7)	22 (11.0)	24 (12.0)	12 (6.0)	142 (71.0)	
**Number of Medical conditions**						
None	27 (3.1)	8 (29.6)	2 (7.4)	5 (18.5)	12 (44.5)	.124
1–2	330 (37.5)	89 (27.0)	62 (18.8)	17 (5.2)	162 (49.0)	
≤ − 3	523 (59.4)	150 (28.7)	89 (17.0)	44 (8.4)	240 (45.9)	
**Physical activity**						
Active	700 (79.5)	177 (25.3)	138 (19.7)	54 (7.7)	331 (47.3)	.000
Inactive	180 (20.5)	70 (38.9)	15 (8.3)	12 (6.7)	83 (46.1)	

*P* from Pearson’s Chi square

According to multinomial logistic regression models (Table 2), the probability of a participant aged 70–79 years making 6–11 doctor visits with respect to a participant who did not visit a doctor in the past year was 5.49 times higher than that of a participant aged 80 years and above. Also, the probability of a participant aged 70–79 years making 12 or more doctor visits with respect to a participant who did not visit a doctor in the past year was 2.03 times higher than that of a participant aged 80 years and above. Although higher ORs were found in all three categories of doctor visits of those aged 60–69 years compared to those who did not visit a doctor in the last year (ORs: 2.18, 2.37, and 1.38), no significant results were obtained.

**Table 2:** Odds ratios from multinomial logistic regression of factors associated with frequency of doctor visits per year (*n* = 880)

Variables	1–5 times doctor visits OR ^**a**^ (95% CI) ^**b**^ P ^**c**^			6 to 11 times doctor’s visits per year OR ^**a**^ (95% CI) ^**b**^ P ^**c**^			More than 12 times OR ^**a**^ (95% CI) ^**b**^ P ^**c**^		
	Model 1a	Model 2a	Model 3a	Model 1b	Model 2b	Model 3b	Model 1c	Model 2c	Model 3c
**SRH**									
Very good or good	0.30 (0.15–0.60) ^***^	0.25 (0.12–0.51) ^***^	0.21 (0.10–0.44) ^***^	0.25 (0.10–0.61) ^***^	0.22 (0.09–0.55) ^***^	0.21 (0.08–0.53) ^***^	0.16 (0.09–0.26) ^***^	0.14 (0.08–0.24) ^***^	0.15 (0.09–0.26) ^***^
Fair	0.75 (0.39–1.42) ^ns^	0.71 (0.37–1.37) ^ns^	0.62 (0.32–1.21) ^ns^	0.62 (0.28–1.38) ^ns^	0.61 (0.27–1.35) ^ns^	0.57 (0.25–1.30) ^ns^	0.21 (0.12–0.35) ^***^	0.21 (0.12–0.35) ^***^	0.22 (0.13–0.37) ^***^
Poor	1 (reference)	1 (reference)	1 (reference)	1 (reference)	1 (reference)		1 (reference)	1 (reference)	1 (reference)
**Physical activity**									
Inactive		0.23 (0.12–0.42) ^***^	0.21 (0.11–0.40) ^***^		0.47 (0.23–0.94) ^*^	0.43 (0.21–0.88) ^**^		0.52 (0.35–0.77) ^***^	0.53 (0.35–0.80) ^***^
Active		1 (reference)	1 (reference)		1 (reference)	1 (reference)		1 (reference)	1 (reference)
**Age (years)**									
60–69			2.18 (0.84–5.66) ^ns^			2.37 (0.50–1.21) ^ns^			1.38 (0.70–2.73) ^ns^
70–79			2.29 (0.88–5.95) ^ns^			5.49 (1.20–0.04) ^**^			2.03 (1.03–3.99) ^**^
80 and over			1 (reference)			1 (reference)			1 (reference)
**Gender**									
Female			0.48 (0.29–0.77) ^***^			0.48 (0.26–0.89) ^**^			1.25 (0.83–1.88) ^ns^
Male			1 (reference)			1 (reference)			1 (reference)
**Education**									
Below Primary education			0.35 (0.14–0.86) ^*^			0.11 (0.02–0.58) ^***^			0.80 (0.38–1.68) ^ns^
Secondary education			0.42 (0.21–0.82) ^**^			0.48 (0.21–1.12) ^ns^			0.76 (0.41–1.43) ^ns^
Tertiary education			1 (reference)			1 (reference)			1 (reference)

No consultation with a doctor in the last 12 months is the reference category for model; ^a^ *OR* Odd Ratio, ^b^ *CI* Confidence Interval, ^c^ p value, *SRH* Self-rated Health; 1 = Reference category for the variable; *P* ^*^ = < 0.05; *p* ^**^ = < 0.01, *p* ^***^ = < 0.001; *ns* Not significant

Females were more likely than males to make doctor visits; about 52% of females compared to 33% of males had made 12 or more doctor visits in the last year (Table 1). The probability of a female participant making 1–5 doctor visits compared to a participant who did not visit a doctor in the past year was 0.48 times lower than that of a male participant (Table 2). The probability of a female participant making 6–11 doctor visits compared to a participant who did not visit a doctor in the last year was 0.48 times lower than that of a male participant. There was no significant gender difference in OR in 12 or more doctor visits compared to no doctor visits in the last 12 months.

Older people studied only up to primary level were more likely to make doctor visits than others (Table 1). The probability of a participant who had studied only at primary level making 1–5 doctor visits compared to a participant who did not visit a doctor in the past year was 0.35 times lower than that of a participant who had studied up to tertiary level. The probability of a participant who had studied at secondary level making 1–5 doctor visits compared to a participant who did not visit a doctor in the past year was 0.42 times lower than that of a participant who had studied beyond secondary level. The probability of a participant who had studied up to primary level making 6–11 doctor visits compared to a participant who did not visit a doctor in the past year was 0.11 times lower than that of a participant who had studied beyond secondary level (Table 2).

#### Enabling factors

Physical activity was significantly associated with the number of doctor visits. A higher percentage of participants who were physically inactive (38.9%) compared to those who were physically active (25.3%) had made zero doctor visits in the past 12 months (*p* < 0.052) (Table 1). The probability of a participant who was physically inactive making 1–5 doctor visits compared to a participant who did not visit a doctor in the past year was 0.21 times lower than that of a participant who was physically active. The probability of a participant who was physically inactive making 6–11 doctor visits compared to a participant who did not visit a doctor in the past year was 0.43 times lower than that of a participant who was physically active. The probability of a participant who was physically inactive making 12 or more doctor visits compared to a participant who did not visit a doctor in the past year was 0.53 times lower than that of a participant who was physically active (Table 2). Monthly family income was not significantly associated with the frequency of doctor visits.

#### Need factors

Self-rated health was significantly associated with the number of doctor visits. About 71.0% of those who rated their health status as poor had made 12 or more doctor visits in the previous year and the corresponding figure for those who rated their health status as good or very good was 41.0% (*p* < 0.051) (Table 1).

The probability of a participant with very good or good SRH making 1–5 doctor visits compared to a participant who did not visit a doctor in the last year was 0.21 times lower than that of a participant with poor SRH. The probability of a participant with very good or good SRH making 6–11 doctor visits compared to a participant who did not visit a doctor in the past year was 0.21 times lower than that of a participant with poor SRH. The probability of a participant with very good or good SRH making 12 or more doctor visits compared to a participant who did not visit a doctor in the past year was 0.15 times lower than that of a participant with poor SRH. Finally, the probability of a participant with fair SRH making 12 or more doctor visits compared to a participant who did not visit a doctor in the past year was 0.22 times lower than that of a participant with poor SRH (Table 2). The number of chronic diseases, however, was not significantly associated with the frequency of doctor visits.

## Discussion

Around three-fourths of the participants had made at least one doctor visit in the past 12 months, with an average of 6.70 visits per year. The results are congruent with other studies [20–22], for example, in Brazil, 83.0% of those aged 50 years and above had made at least one doctor visit in the past 12 months.

Age, gender, educational level, SRH, and physical activity were found to be associated with the frequency of doctor visits. The participants aged 80+ years and those who had studied only up to primary level were more likely to have made infrequent doctor visits in the past year. Women were more likely than men to make at least one doctor visit per month. Those who rated their health as poor and those who were sufficiently physically active have a tendency to make a higher number of doctor visits than others after adjusting for age, gender, and educational level.

A previous study conducted in Sri Lanka [23] reported that age has no influence on healthcare use patterns in older adults. However, many research findings indicate that individuals are likely to reduce their healthcare utilization as they become older [24, 25]. Although positive relationships between age and healthcare demand have also been found [26]. Older adults expect healthcare to be comprehensive, desiring psychological and emotional care more than physical attention [27]. Those who are in the 80+ years age group may need assistance to access healthcare and deficiencies in providing required assistance may obstruct the oldest-old from seeking the required healthcare. Results indicated that participants aged 70–79 years tended to make more doctor visits than those aged 60–69 years, probably because advanced age is accompanied by poorer health conditions. However contextual and country-specific factors that would probably interfere in this relationship need further investigations.

Older women compared to older men were more likely to make frequent doctor visits. It has been observed that compared to men, women presented with a greater number of chronic diseases and were less likely to be independent in activities of daily living [28–31]. A higher value in BMI in women probably would indicate their vulnerability to chronic diseases such as hypertension and diabetes. In the literature, it has been shown that gender is a strong predisposing factor that influences healthcare utilization, and females are more likely than males to seek healthcare, especially in their later life [16, 26]. In Sri Lanka, females outlive males, and older women experience greater ill health and loss of activities of daily living as they age [23]. Further, older women, generally do not have a fixed income and are dependent on others for a living [23]. A study conducted in Sri Lanka indicated that expenditure on consultations and drugs and accessibility issues were crucial determinants of demand for healthcare in women [23]. In 2014, male and female labor force participation rates in Sri Lanka were 74.6 and 34.7%, respectively. Women in many low and middle-income countries are likely to be illiterate, unemployed, widowed, and dependent on others as well as having worse perceptions of health [32, 33]. The lack of an appointment system in most hospitals, older women with low educational levels are more likely than others to make doctor visits because they do not have formal occupation, leaving them with more free time to visit public health free clinics during the day. Moreover, the issuance of special identity cards for older people in Sri Lanka may also motivate older women to use free healthcare services frequently.

It was observed that participants with higher educational level (tertiary and above) were likely to make doctor visits more frequently than others. Educated older adults may have higher health literacy and family income, resulting in better awareness about health conditions and a higher perceived severity of illness [28, 34].

Family income and multiple chronic conditions, which have been found to be associated with frequency of doctor visits in older adults in other countries [7, 35], were not significantly associated with frequency of doctor visits in this target population. Sri Lankans enjoy state-funded free universal healthcare that ensures equity in healthcare access irrespective of socioeconomic status. The public healthcare system in Sri Lanka is decentralized and easily accessible across the country. There is a well-established hospital network, and referrals are often made for severe cases free of travelling cost. These issues may have minimized accessibility and affordability issues in healthcare services for older adults [36]. Although a positive relationship between number of comorbidities and use of healthcare services has been observed in many studies [37, 38], our data do not support this relationship. In Sri Lanka, there have been recent changes to the primary care curative health system to tackle the non-communicable disease burden [5, 23]. A study conducted in Sri Lanka reported that 48.0% of the respondents in the study had visited clinics on a regular basis [33]. Emerging chronic health challenges in older adults are also dealt with by these facilities free of charge. Thus, irrespective of the number of health conditions, older adults tend to make monthly clinic visits to consult a doctor and receive the required treatment.

In the current study, physically inactive older adults were less likely to make frequent doctor visits than their counterparts. People are inclined to lead sedentary lives as they age, increasing their vulnerability to chronic diseases and disabilities. Although it is expected that physically active older adults would use fewer healthcare services because they are less likely to develop chronic diseases [23], the opposite was observed in our study. In many past studies, physical activity or exercise behavior has been considered a predisposing factor [24] or a need factor [39]. However, physical activity-related skills, such as driving ability, can be an enabling factor for doctor visits. So here we have categorized physical activity as an enabling factor because we were attempting to identify the role of physical fitness as a form of moral support for older adults going for health advisers within close proximity. Low muscle strength is central to geriatric physical disabilities [40], which is related to functional limitations including mobility. On the contrary, it can be postulated that individuals who are more conscious of their physical health tend to maintain a physically active lifestyle and use healthcare services more often. Thus, the influence of physical strength and balance in healthcare seeking behaviors of older adults needs further investigations.

Older adults with poor SRH had significantly higher odds of visiting doctors independent of age, gender, and physical activity status (sufficiently physically active or not). Older adults are more likely than other age groups to rate their SRH as fair or poor [41]. A prominent feature of our results is the consistency of the association between poor SRH and different categories of doctor visits. Self-rated health could be a simple indicator and an effective marker to plan healthcare resource allocation and model psychosocial support services for older adults who seek repeated medical consultations. It is noteworthy that SRH has been found to be associated with income and social interactions [30]. There is a growing appreciation that SHR is a broad construct related to the self-concept of health rather than a reflection of medical health status [42].

Several studies have confirmed the associations between physical activity, SRH, socioeconomic status, and utilization of healthcare [35, 37, 40]. Some studies have shown that SRH does not mediate the association between physical activity and healthcare utilization [13]. The reason may be that fatigue and stress, common in later life, are of diminishing relevance for SRH with increasing age. Previous studies have also shown that the strongest predictors of healthcare utilization are need factors, followed by enabling and predisposing factors [9]. Similarly, in our study, SRH had the strongest association with doctor visits in urban-dwelling Sri Lankan older adults; those with poor SRH tended to use more healthcare services. Physical activity, a proxy of physical fitness, is the next important factor related to number of doctor visits. Those who are physically active tend to have good physical fitness and fewer limitations in activities of daily living, and can, thus, visit doctors by themselves. Physical activity should be encouraged among the oldest-old, the most vulnerable to poor physical and mental health status, to guarantee equity in healthcare for all segments of older adults in the country.

There are several limitations in this study. Cross-sectional nature of the study design did not allow us to reveal cause-effect relationships. Limited number of enabling and need factors was considered in the study and it is a limitation. The study use number of doctor visit as proximity to study healthcare utilization.. Self-rated health and physical activity behavior can potentially be used as screening tools for the identification of older adults who are likely to make inadequate number of doctor visits. Further longitudinal studies that are taken into consideration other enabling and need factors of doctor visits are needed to make a comprehensive picture of correlates of doctor visits by older-adults in Sri Lanka.

## Conclusions

In this study, some selected predisposing, enabling, and need factors associated with doctor visits, an indicator of healthcare utilization, in Sri Lankan older adults were examined. Self-rated health was negatively association with doctor visits. Physical activity is positively contributed to make doctor visits, indicating its importance with regard to equity in healthcare access for older adults. Gender and age are the most important predisposing factors in doctor visits, an important indicators of healthcare utilization in older adults; older men and those of aged 80+ years have a tendency to make the least frequent doctor visits. Men, those aged 80+ years and physically inactive older adults, whose health care needs remain unmet, need to be motivated for proper use of health care facilities.

The patterns of doctor visits and their correlates identified in this study would be useful in restructuring geriatric healthcare services in Sri Lanka. Oldest-old and physically inactive older adults need a grass root level service provider to enhance proper healthcare utilization. The study results suggest that there is a necessity of a direct healthcare worker, someone who is assigned specifically to care for older adults such as a community geriatric care nurse, who would be responsible for providing care, monitoring health conditions, and making the necessary referrals for older adults living in urban settings. Geriatric nursing management and healthcare services in the country should be facilitated to identify and provide necessary care for vulnerable groups suggested in this study.

## Supplementary Information

**Additional file 1: Supplementary Data File.** Doctor visits of older Sri Lankans.

## Data Availability

The data sets and other study materials are available for those who make reasonable request. Please contact Dr. Bilesha Perera (email: bileshap@gmail.com).

## Abbreviations

SRH: Self rated health
OR: Odds ratio
